# A Glucose-Responsive Hydrogel with Multifunctional Properties for Accelerated Diabetic Wound Healing

**DOI:** 10.34133/bmr.0405

**Published:** 2026-08-19

**Authors:** Zhaoguo Wang, Susu Lei, Feng Lai, Chao Wang, Jing Xu, Shulan Xu

**Affiliations:** ^1^ Center of Oral Implantology, Stomatological Hospital, School of Stomatology, Southern Medical University, Guangzhou 510632, China.; ^2^ School of Biomedical Engineering, Shanghai Jiao Tong University, Shanghai 200240, China.; ^3^ College of Animal Science and Technology, Inner Mongolia Minzu University, Tongliao 028000, China.

## Abstract

Diabetic wounds are characterized by oxidative stress, chronic inflammation, and impaired tissue regeneration under persistent hyperglycemic conditions. Herein, we report an injectable dual-dynamic covalent hydrogel fabricated from phenylboronic-acid-functionalized oxidized sodium alginate and gallic-acid-conjugated chitosan. Crosslinked via reversible Schiff base and boronate ester bonds, the hydrogel exhibits excellent injectability, self-healing capability, and structural stability. Under hyperglycemic conditions, competitive glucose binding modulates the boronate ester equilibrium and induces glucose-responsive release of galloyl-containing species. These glucose-responsive release behaviors contribute to the antioxidant, antibacterial, and immunoregulatory activities of the hydrogel. In vitro and in vivo results demonstrate that the hydrogel promotes macrophage polarization toward the anti-inflammatory M2 phenotype, alleviates inflammatory responses, enhances angiogenesis, and accelerates skin regeneration. Collectively, the phenylboronic-acid-functionalized oxidized sodium alginate and gallic-acid-conjugated chitosan hydrogel represents a multifunctional glucose-responsive biomaterial with considerable potential for diabetic wound therapy.

## Introduction

Diabetic wounds (DWs) represent one of the most prevalent and severe complications of diabetes, affecting roughly 15% to 25% of patients worldwide [1]. Persistent hyperglycemia leads to excessive accumulation of reactive oxygen species (ROS), causing microvascular damage, peripheral neuropathy, immune imbalance, and sustained oxidative stress and inflammation [2]. Within this pathological state, DWs typically exhibit delayed healing, elevated susceptibility to infection, and high recurrence, and severe cases may progress to tissue necrosis, amputation, or death [3]. Conventional dressings, including gauze and foam materials, primarily act as passive physical barriers. They lack the capacity to sense or adjust to the changing wound microenvironment and cannot provide controlled drug release, which restricts their therapeutic effectiveness [4].

Recently, functional hydrogels have emerged as promising biomaterials for DW treatment owing to their excellent biocompatibility, high water content, interconnected porous structure, and tunable physicochemical properties [5]. Various functional hydrogels, including photothermal-responsive [6], probiotic-composite [7], high-toughness [8], and dual-dynamic covalent hydrogels [9,10], have been developed to improve wound repair by responding to changes in the wound microenvironment. Among natural polymers, chitosan (CS) and sodium alginate (SA) are widely used for hydrogel fabrication because of their excellent biocompatibility, biodegradability, and low immunogenicity [11]. SA readily forms stable hydrogel networks, whereas CS possesses intrinsic antibacterial activity and promotes cell adhesion and proliferation. However, conventional natural polymer hydrogels generally suffer from insufficient mechanical strength, limited bioactivity, and inadequate responsiveness to the DW microenvironment, which restricts their therapeutic performance [12].

To overcome these limitations, chemically modified natural polymers have been widely explored for constructing multifunctional stimuli-responsive biomaterials. Among various physiological stimuli, hyperglycemia is a defining feature of DWs, making glucose-responsive drug delivery an attractive therapeutic strategy [13]. Phenylboronic acid (PBA), a well-established glucose-recognition moiety, reversibly interacts with *cis*-diol-containing molecules through dynamic boronate ester bonds and has therefore been extensively employed in glucose-responsive biomaterials [14]. As glucose concentration increases, glucose competitively interacts with PBA, shifting the dynamic boronate ester equilibrium and facilitating glucose-responsive therapeutic release [15]. Xu et al. [16] developed a glucose-responsive hydrogel based on reversible boronate ester interactions between PBA-modified hyaluronic acid methacrylate and catechin, which accelerated DW healing. However, most PBA-based hydrogel systems still face challenges in balancing structural stability and sustained therapeutic release under prolonged hyperglycemic conditions because continuous glucose stimulation can gradually shift the reversible boronate ester equilibrium, thereby affecting the integrity of the dynamic crosslinking network [17]. Therefore, incorporating a multifunctional *cis*-diol-containing component that can participate in the dynamic boronate ester network while simultaneously providing therapeutic bioactivity represents an attractive strategy for improving glucose-responsive hydrogels.

In this regard, gallic acid (GA), a naturally occurring polyphenol, has attracted increasing attention for chronic wound repair because of its antioxidant, antibacterial, and anti-inflammatory activities [18]. Structurally, the *ortho*-trihydroxyphenyl groups of GA serve as *cis*-diol ligands capable of reversibly interacting with PBA, while the abundant phenolic hydroxyl groups contribute to network stabilization through hydrogen bonding [19]. In addition, GA has been reported to promote macrophage polarization from the pro-inflammatory M1 phenotype toward the reparative M2 phenotype, thereby alleviating chronic inflammation and improving tissue regeneration [20–22]. These unique physicochemical and biological properties make GA an attractive functional component for glucose-responsive hydrogels.

Based on these considerations, we developed an injectable dual-dynamically covalent crosslinked hydrogel (OSA-PBA/CS-GA) by integrating glucose-responsive boronate ester interactions with the multifunctional bioactivity of GA. The hydrogel combines excellent injectability, self-healing capability, structural stability, and glucose-responsive release behavior. Under hyperglycemic conditions, competitive glucose binding modulates the dynamic boronate ester equilibrium, resulting in partial relaxation of the hydrogel network and facilitating the release of galloyl-containing species. The glucose-responsive release of these galloyl-containing species is expected to contribute to the antioxidant, antibacterial, and anti-inflammatory functions of the hydrogel. Moreover, the hydrogel modulates the wound immune microenvironment by promoting macrophage polarization toward the reparative M2 phenotype, thereby alleviating chronic inflammation, enhancing angiogenesis, and facilitating DW repair. Collectively, this work presents a multifunctional glucose-responsive hydrogel that integrates adaptive network dynamics with therapeutic bioactivity for DW treatment.

## Materials and Methods

### Materials and reagents

SA, CS, 3-aminophenylboronic acid (APBA), glutaraldehyde, 1-(3-dimethylaminopropyl)–3-ethylcarbodiimide hydrochloride (EDC), 2-(*N*-morpholino) ethanesulfonic acid, and *N*-hydroxysuccinimide (NHS) were all purchased from Shanghai Macklin Biochemical Technology Co., Ltd (China). Other chemicals were of analytical grade, and ultrapure water (Milli-Q system, Germany) was used for solution preparation.

### Preparation of OSA-PBA/CS-GA

#### Synthesis of OSA

OSA synthesis was carried out following an optimized reported method [23]: 5.0 g SA was dissolved in 100 mL of ethanol–water (1:1, v/v), and the pH was adjusted to 3.0. Sodium periodate (1:1 molar ratio to SA) was added, and the reaction was stirred for 6 h in the dark. Then, 3 mL of ethylene glycol was added to terminate the reaction. The solution was dialyzed [molecular weight cutoff (MWCO) = 3,500 Da] for 72 h (water changed twice daily) and freeze-dried for 72 h to obtain white spongy OSA.

#### Synthesis of OSA-PBA

Of OSA, 1.0 g was dissolved in 100 mL of water. EDC·HCl (0.96 g) and NHS (0.58 g) were added at pH 6.0 and stirred for 30 min for activation. Then, 0.39 g of 3-aminophenylboronic acid was added, and the mixture was stirred for 24 h at 10 °C under N_2_. The product was dialyzed (MWCO = 3,500 Da) for 72 h (water changed twice daily) and vacuum freeze-dried to obtain OSA-PBA.

#### Synthesis of CS-GA

Of GA, 1.0 g was dissolved in 20 mL of absolute ethanol; 1.19 g EDC·HCl and 0.71 g NHS were added and activated for 30 min. Separately, 1.00 g CS was dissolved in 1.0% (v/v) acetic acid (80 mL, pH 4.5). Activated GA was added dropwise into CS, and the mixture reacted for 6 h at 25 °C in the dark (tin foil wrapped). The solution was dialyzed (MWCO = 3,500 Da) for 72 h (water changed twice daily) and freeze-dried at –60 °C for 72 h to obtain fluffy CS-GA.

#### Preparation of OSA-PBA/CS-GA

Of OSA-PBA, 400 mg and 400 mg CS-GA were dissolved in 10 mL of water (4%, w/v). According to V (OSA-PBA solution):V (CS-GA solution) = 2:1, 1:1, and 1:2, the mixtures were vortexed for 1 min to allow complete crosslinking and left to stand at 37 °C for 0.5 h, producing OSA-PBA/CS-GA hydrogels with different crosslinking degrees named OSA-PBA/CS-GA_1_, OSA-PBA/CS-GA_2_, and OSA-PBA/CS-GA_3_.

### Structural characterization methods

Ultraviolet (UV) spectra: Agilent Cary 3500 spectrophotometer. SA, OSA, OSA-PBA, GA, CS, and CS-GA solutions were prepared and scanned from 200 to 400 nm, and their spectra were recorded. Fourier-transform infrared spectra: Nicolet 6700 spectrometer. After drying, samples were mixed with KBr (1:100, w/w), ground, pressed into slices, and scanned from 4,000 to 500 cm^–1^. ^1^H nuclear magnetic resonance spectra: Bruker Avance III (600 MHz). D_2_O was used to dissolve samples, and tetramethylsilane served as the internal standard. Scanning electron microscopy (SEM): Navigator-100. Hydrogel samples were dried in a freeze dryer, attached to the sample stage with conductive glue, and gold sprayed. The microstructure was observed under an accelerating voltage of 5 kV.

### Performance testing

Mechanical property testing: Cylindrical hydrogels (diameter 10 mm; height 6 mm) were placed on the test bench for uniaxial testing at room temperature using an electronic universal testing machine (Gotech Al-3000-U). The compression ratio was set to 80%, and the compression rate was 20.0 mm/min. For this hydrogel, the slope of the first 10% linear region of the stress–strain curve was taken as its Young’s modulus.

Swelling property testing: Hydrogel swelling behavior was assessed by weighing. Hydrogels were weighed to record initial mass (*m*_0_), then immersed in phosphate-buffered saline (PBS) and incubated at 37 °C with oscillation (50 r/min). Samples were collected at 1, 2, 3, 4, 6, 10, 12, 14, and 16 h, and surface moisture was blotted dry before weighing to obtain wet mass (*m*_t_). The swelling ratio was calculated as swelling ratio = [(*m_t_* − *m*_0_)/*m*_0_] × 100%. The average swelling ratio of each group was determined, and a swelling ratio–time curve was plotted.

Glucose responsiveness testing: 0.2-mL hydrogel samples were weighed to record initial mass (*m*_0_), then placed in 20 mL PBS with different pH values and glucose concentrations (0, 5.6, and 11.1 mmol/L). All samples were incubated with oscillation (50 r/min) at 37 °C. Samples were collected at specific time points, and surface moisture was blotted dry before weighing to record wet mass (*m*_w_). The swelling ratio was calculated as follows: swelling ratio = (*m*_w_/*m*_o_) × 100%.

Rheological property testing: Hydrogels (diameter 10 mm; height 3 mm) were placed on the test bench, and rheological properties were measured using a rotational rheometer (HAAKE Mars 40) at 25 °C. Amplitude scanning: frequency 1 rad/s, amplitude 1% to 1,000%. Frequency scanning: strain amplitude 1% to 100%. Alternating strain scanning: Hydrogels were measured at 1% and 200% strain at 1.0 rad/s.

In vitro degradation assay: The in vitro degradation of OSA/CS, OSA-PBA/CS, and OSA-PBA/CS-GA hydrogels was assessed by incubating pre-weighed samples in PBS (pH 7.4) at 37 °C. At predetermined time points, the samples were retrieved, gently blotted to remove excess buffer, and weighed to determine the remaining mass, which was calculated as the percentage of the initial weight.

Glucose-responsive release assay: Glucose solutions (5.6 and 11.1 mM) were prepared in 0.01 M PBS (pH 7.4), with glucose-free PBS as a control. Then, 1.0 mL hydrogel was immersed in 10.0 mL medium and shaken at 37 °C, 50 rpm. At scheduled time points (30 to 1,440 min), 500 μL medium was collected and replenished with an equal fresh buffer. Released galloyl species were quantified by UV absorbance at 298 nm. All tests were performed in triplicate. Cumulative release (CR, %) was calculated as

$$\mathrm{CR}\;\left(\%\right)=\frac{10\times C_{n}+0.5\times \sum_{i=1}^{n-1}C_{i}}{m_{\text{total}}}\times 100$$ (1)

where *C_n_* = galloyl concentration at the *n*-th sampling and $m_{\text{total}}$ = total galloyl mass loaded in hydrogel. Coefficients 10 and 0.5 correspond to total medium volume (10.0 mL) and sampling volume (0.5 mL), respectively.

### Competitive binding experiments

To determine the binding constant between PBA and Alizarin Red S (ARS) (denoted as *K*_ARS_), a 100-μM ARS solution was prepared in 0.1 M PBS (pH 7.4). ARS concentration was fixed at 50 μM, gradient OSA-PBA was added to obtain PBA concentrations ranging from 0 to 1,000 μM at 37 °C with a total volume of 2 mL and then equilibrated for 15 min, and fluorescence intensity was detected. The binding constant *K*_ARS_ was calculated using the Benesi–Hildebrand fitting method [24]. In competitive displacement assays, the equilibrium binding constants (*K*_eq_) of GA, OSA, and glucose with PBA were determined. Briefly, a mixed solution containing 50 μM ARS and 100 μM OSA-PBA was balanced for 15 min to record initial fluorescence. GA, OSA, and glucose (0–50 mM) were gradually added separately with volume change below 2%. Fluorescence intensity was detected after 15 min of equilibration, and competitive curves were plotted and fit to acquire apparent binding constants via the nonlinear least-squares method [25].

### Cytotoxicity evaluation

L929 and RAW264.7 were obtained from Procell (Wuhan, China). Human umbilical vein endothelial cells (HUVECs) were donated by Dr Yaya Bai from Shanghai Jiao Tong University and acquired from the National Collection of Authenticated Cell Cultures (Shanghai, China). L929 cells were cultured in Dulbecco’s Modified Eagle Medium (DMEM; Gibco, USA) containing 10% fetal bovine serum (FBS; Gibco) and 1% penicillin/streptomycin (p/s; Gibco), HUVECs were cultured in extracellular matrix (ECM; Gibco) containing 10% FBS and 1% p/s, whereas RAW264.7 cells were maintained in Alpha Minimum Essential Medium (Gibco) containing the same concentrations of 10% FBS and 1% p/s. Cells were incubated at 37 °C, 95% humidity, and 5% CO_2_.

To assess cytotoxicity, cells were co-cultured with OSA/CS, OSA-PBA/CS, and OSA-PBA/CS-GA hydrogels using the Live/Dead Cell Staining Kit (Beyotime, China). Specifically, L929, RAW264.7, and HUVECs (5,000/well) were plated in 96-well plates, and each hydrogel was co-cultured at 37 °C for 72 h. Calcein acetoxymethyl ester/propidium iodide (PI) staining working solution was prepared according to kit instructions and added for incubation at 37 °C, protected from light for 30 min, followed by imaging with a fluorescence microscope (DMi8; Leica, Germany). Live and dead cells emitted green (calcein acetoxymethyl ester) and red (PI) fluorescence, respectively. For cell viability analysis, hydrogel extracts of OSA/CS, OSA-PBA/CS, and OSA-PBA/CS-GA were prepared as previously reported [26], with minor modifications. Briefly, the obtained extracts were filtered through a 0.22-μm membrane filter. Then, 100 μL of each extract was co-cultured with the same cells at the same density at 37 °C for 24 h and 72 h, respectively. Cell viability was determined using a Cell Counting Kit-8 (Solarbio, China), and the absorbance at 450 nm was measured using a Tecan microplate reader (Tecan, Switzerland).

### Cell scratch assay

HUVECs were used as a model to evaluate how hydrogels influenced cell scratch behavior. HUVECs (2.5 × 10^5^/well) were seeded into 6-well plates and cultured until cell adherence reached 95% to form a confluent monolayer. A 200-μL pipette tip was used to create a straight scratch to simulate a wound. HUVECs were co-cultured with OSA/CS, OSA-PBA/CS, and OSA-PBA/CS-GA hydrogels, treated with 100 μM H_2_O_2_ for 2 h, and then maintained in 10 μM H_2_O_2_, followed by 24 h of continuous culture. Cell migration was then observed and photographed using a microscope (Leica).

### Tube formation assay

The Matrigel tube formation assay was used to evaluate how hydrogels influenced the tube-forming capacity of HUVECs. Twenty microliters of Matrigel (356234; Corning, USA) was added to each well of a 24-well plate and incubated for 30 min to allow solidification. HUVECs (1.5 × 10^5^/well) were then seeded onto the solidified Matrigel. HUVECs were co-cultured with OSA/CS, OSA-PBA/CS, or OSA-PBA/CS-GA hydrogels in the corresponding wells, treated with 100 μM H_2_O_2_ for 2 h, and maintained in 10 μM H_2_O_2_. After 6 h of culture, images were captured by a microscope (Leica), and tubular structures were analyzed using ImageJ software with an angiogenesis plugin.

### Antioxidant assay

To assess how hydrogels affected H_2_O_2_-induced oxidative stress, HUVECs were co-cultured with OSA/CS, OSA-PBA/CS, or OSA-PBA/CS-GA hydrogels in separate wells, treated with 100 μM H_2_O_2_ for 2 h, and maintained in 10 μM H_2_O_2_ for 24 h. 2′,7′-Dichlorodihydrofluorescein diacetate probe (E004-1-1; Nanjing Jiancheng, China) was added and incubated for 20 min in the dark. After PBS washes to remove excess probe, fluorescence microscopy (Leica) was used for image capture. Cells were washed twice with PBS, resuspended, and subjected to flow cytometric analysis (Beckman, Germany). In addition, hydrogels were evaluated for in vitro antioxidant capacity using 2,2-diphenyl-1-picrylhydrazyl (A153-1-1; Nanjing Jiancheng) and 2,2′-azino-*bis*(3-ethylbenzothiazoline-6-sulfonic acid (A015-2-1; Nanjing Jiancheng) assays. To further explore the antioxidant mechanisms, superoxide dismutase (SOD) and catalase (CAT) activities, along with malondialdehyde (MDA) levels, were measured. These indicators were assayed strictly according to the corresponding kit protocols (SOD, CAT, and MDA Assay Kits; Nanjing Jiancheng).

### Antibacterial assay

To evaluate the in vitro antibacterial performance of the prepared hydrogels, *Escherichia coli* (ATCC25922) and *Staphylococcus aureus* (ATCC25923) were selected as representative bacterial strains. Luria–Bertani media supplemented with 5.6 mmol/L glucose or 11.1 mmol/L glucose were prepared separately, and all antibacterial tests were performed in parallel under the 2 glucose concentrations. Briefly, 200 μL of each hydrogel sample was preloaded into culture vessels, followed by the addition of bacterial suspension at a concentration of 1 × 10^5^ colony-forming units/mL. The mixtures were incubated at 37 °C for 12 h. After incubation, serial dilutions of bacterial suspensions were prepared and spread onto agar plates, which were further cultured at 37 °C for 20 h before colony enumeration. Colony images were captured using an automatic colony counter (Scan 500; Interscience, France), and morphological alterations of bacteria were visualized via a Navigator-100 scanning electron microscope. For live/dead bacterial staining, bacterial pellets were incubated with dimethylacridine orange/PI dual staining solution at room temperature in the dark for 15 min. Fluorescent signals were detected under a Leica confocal laser scanning microscope, where green fluorescence indicated live bacteria and red fluorescence represented dead bacteria.

### Flow cytometry analysis

RAW264.7 cells (2 × 10^5^/well) were seeded into 6-well plates and cultured at 37 °C, 5% CO_2_ until 80% confluent. Cells were then co-cultured with OSA/CS, OSA-PBA/CS, or OSA-PBA/CS-GA hydrogels, with 200 ng/mL lipopolysaccharide added for activation. After 24 h, cells were collected, trypsinized, centrifuged, and resuspended prior to fixation with 4% paraformaldehyde. After 0.1% Triton X-100 permeabilization and 5% bovine-serum-albumin blocking (30 min, room temperature), cells were incubated overnight at 4 °C with antibodies against CD86 (Biotin-FcA65673) and CD206 (98031-1-RR) (Proteintech, China), followed by 1-h incubation with fluorescent secondary antibody (111-165-003; Jackson ImmunoResearch, USA) at room temperature in the dark. Finally, cells were resuspended in PBS, and CD86/CD206 expression was analyzed by flow cytometry (Beckman Coulter, USA).

### Immunofluorescence staining

Cell seeding, lipopolysaccharide stimulation, and hydrogel co-culture were performed in strict accordance with the methods described in “Flow cytometry analysis” section. After 24 h of treatment, the cells were fixed, permeabilized, and blocked. Subsequently, the cells were incubated with primary antibodies against CD86 (catalog no. 30691-1-AP) and CD206 (catalog no. 98031-1-AP), both purchased from Proteintech. After that, the cells were incubated with matched fluorescent secondary antibodies (Proteintech). The cell nuclei were counterstained with 4′,6-diamidino-2-phenylindole, and the expression of M1/M2 phenotypic markers was observed using a Leica STELLARIS 5 confocal microscope (Leica).

### DW rat model construction

The Animal Ethics Committee of Southern Medical University approved all animal care and experiments (Approval No. NYKQ-LAEC-2025-019). Eight-week-old specific-pathogen-free male Sprague–Dawley rats were obtained from GDMLAC (Guangdong, China) and acclimatized under standard laboratory conditions for 1 week before experiments. After a 12-h fast, diabetes was induced via intraperitoneal injection of streptozotocin (STZ, 60 mg/kg; Solarbio) dissolved in 0.1 M citrate buffer (pH 4.5; Solarbio). Fasting blood glucose ≥16.7 mmol/L measured consecutively for 7 d indicated successful diabetes induction, with blood samples collected from the tail vein starting 96 h postinjection. Rats were anesthetized by inhalation of isoflurane (RWD, China) in 100% oxygen using an anesthesia machine (Veta1; Mindray Animal Medical, China). After shaving the dorsal hair, iodophor was used for skin disinfection. A sterile biopsy punch created 2 symmetrical full-thickness skin wounds (diameter 10 mm), as reported previously [27,28]. Each wound was incubated with 1 × 10^7^ colony-forming units/mL *S. aureus* suspension (100 μL) for 30 min at room temperature to establish infected wounds. Modeled rats were randomized into 5 groups (negative control, CS, OSA/CS, OSA-PBA/CS, and OSA-PBA/CS-GA) with *n* >6 rats per group. All wounds were dressed with Tegaderm and an elastic bandage, changed every 2 d; rat behavior and intake were monitored. Wound photos were taken at 0, 3, 7, 10, 14, and 17 d postoperation (dpo) under identical conditions. Area (*A_t_*) was measured via ImageJ; wound reduction rate = [(*A*_0_ − *A_t_*)/*A*_0_] × 100% (*A*_0_: original area). At 14 dpo, 5 rats/group were euthanized. Skin tissues were excised: one part was placed in 4% paraformaldehyde, and the other was snap-frozen in liquid nitrogen and stored at –80 °C. Remaining rats were euthanized at 17 dpo for final healing evaluation.

### Histological assay

For histological analysis, skin samples were fixed in 4% paraformaldehyde, dehydrated, paraffin embedded, and sectioned into 5-μm serial slices for hematoxylin–eosin (H&E) and Masson trichrome staining. H&E-stained sections (dewaxed, stained, and neutral gum-sealed) were examined using a digital pathology scanner (Aperio VERSA; Leica) to assess epithelial repair, and ImageJ was used to quantify epithelial thickness. Masson-stained sections (dewaxed and stained according to the kit) showed blue collagen, black nuclei, and red cytoplasm, observed with the same scanner, and ImageJ quantified collagen area ratio to evaluate fibrotic repair.

### Immunohistochemistry staining

Paraffin sections were dewaxed in water, and citric acid buffer was used for antigen retrieval. Then, 3% H_2_O_2_ was added to block endogenous peroxidase activity and nonspecific binding. Sections were incubated overnight at 4 °C with primary antibodies including matrix metalloproteinase 9 (MMP9; A0289), interleukin-6 (IL-6; A21264) (ABclonal, China), CD86 (30691-1-AP), CD206 (18704-1-AP) (Proteintech), CD31 (ab281583), vascular endothelial growth factor (VEGF; ab32152) (Abcam, UK), and alpha smooth muscle actin (α-SMA; A2235, ABclonal). After primary incubation, sections received horseradish-peroxidase-labeled secondary antibody (Solarbio). Color development was performed using 3,3′-diaminobenzidine chromogen (Solarbio), followed by gradient dehydration and neutral gum mounting. The digital pathology scanner (Aperio VERSA; Leica) was used to observe positive expression, and ImageJ software quantified integrated optical density in positive regions.

### Quantitative real-time polymerase chain reaction

Trizol reagent (Takara, Japan) was used to extract total RNA from HUVECs, RAW264.7 cells, and skin tissue samples. RNA content and quality were assessed with a bio-photometer (Thermo Scientific, USA). cDNA was synthesized according to the reverse transcription kit instructions (TRANSGEN, China). Relative target gene expression was determined by quantitative real-time polymerase chain reaction (qRT-PCR), with glyceraldehyde-3-phosphate dehydrogenase used as an endogenous control to correct for inter-sample differences. The 2^–ΔΔCt^ method was used to calculate target gene expression. Table 1 lists the corresponding primer sequences.

**Table 1.** Primer sequences

Species	Genes	Primer forward (5′–3′)	Primer reverse (3′–5′)
Mice	*iNOS*	TGTCGCAGCTCCCTATCTTG	CATTGGCCAGCTGCTTTTGC
Mice	*CD86*	TGCTCATCTATACACGGTTAC	TTTCTTGGTCTGTTCACTCTC
Mice	*MMP9*	CTGGACAGCCAGACACTAAAG	CTCGCGGCAAGTCTTCAGAG
Mice	*IL-6*	CAAAGCCAGAGTCCTTCAGAG	AGCATTGGAAATTGGGGTAG
Mice	*Arg-1*	AAGAATGGAAGAGTCAGTGTGG	GGGAGTGTTGATGTCAGTGTG
Mice	*CD206*	AGCTTCATCTTCGGGCCTTTG	GGTGACCACTCCTGCTGCTTAG
Mice	*IL-10*	TCACTCTTCACCTGCTCCAC	CTATGCTGCCTGCTCTTACTC
Mice	*GAPDH*	TGGCAAAGTGGAGATTGTTGCC	AAGATGGTGATGGGCTTCCCG
Rat	*VEGF*	CCTTAGGTGCTTCCCCATATC	GAGCCCGCATTCTCGTTC
Rat	*α-SMA*	TGCCATGTATGTGGCTATTCA	ACCAGTTGTACGTCCAGAAGC
Rat	*COL1A1*	CATGTTCAGCTTTGTGGACCT	GCAGCTGACTTCAGGGATGT
Rat	*CD31*	CACCGTGATACTGAACAGCAA	GTCACAATCCCACCTTCTGTC
Rat	*IL-1*	ATCTCACAGCAGCATCTCGACAAG	CACACTAGCAGGTCGTCATCATCC
Rat	*IL-6*	AGGAGTGGCTAAGGACCAAGACC	TGCCGAGTAGACCTCATAGTGACC
Rat	*TNF-α*	CACCATGAGCACGGAAAGCA	GCAATGACTCCAAAGTAGACC
Rat	*MMP9*	TGGAAACTCACACGCCAGAAG	AGCCGGGAACGTATCTGGA
Rat	*CD86*	CTCAGTGATCGCCAACTTCA	CTGCATGTTGTCGCCATACT
Rat	*IL-10*	ATTGAACCACCCGGCATCTA	CAACGAGGTTTTCCAAGGAG
Rat	*Arg-1*	GCCTGAGAGTCTGGCCTACA	ATCCCTCAGGCTGCTCCATT
Rat	*CD206*	CCTATGAAAATTGGGCTTACGG	CTGACAAATCCAGTTGTTGAGG
Rat	*GAPDH*	CATGGCCTTCCGTGTTCCTA	GCCTGCTTCACCACCTTCTT
Human	*CD31*	AACAGTGTTGACATGAAGAGCC	TGTAAAACAGCACGTCATCCTT
Human	*VEGF*	AGGGCAGAATCATCACGAAGT	AGGGTCTCGATTGGATGGCA
Human	*GAPDH*	GGAGCGAGATCCCTCCAAAAT	GGCTGTTGTCATACTTCTCATGG

### Statistical analysis

Each assay was performed in triplicate. Results were expressed as mean ± SD. GraphPad Prism 9.5 software was used for statistical analysis. For multiple group comparisons, one-way analysis of variance followed by Tukey post hoc test was used for pairwise comparisons. ^*^P < 0.05, ^**^P < 0.01, ^***^P < 0.001, ^****^P < 0.0001.

## Results and Discussion

### Preparation and physicochemical characterization of OSA-PBA/CS-GA hydrogel

**Fig. 1.**
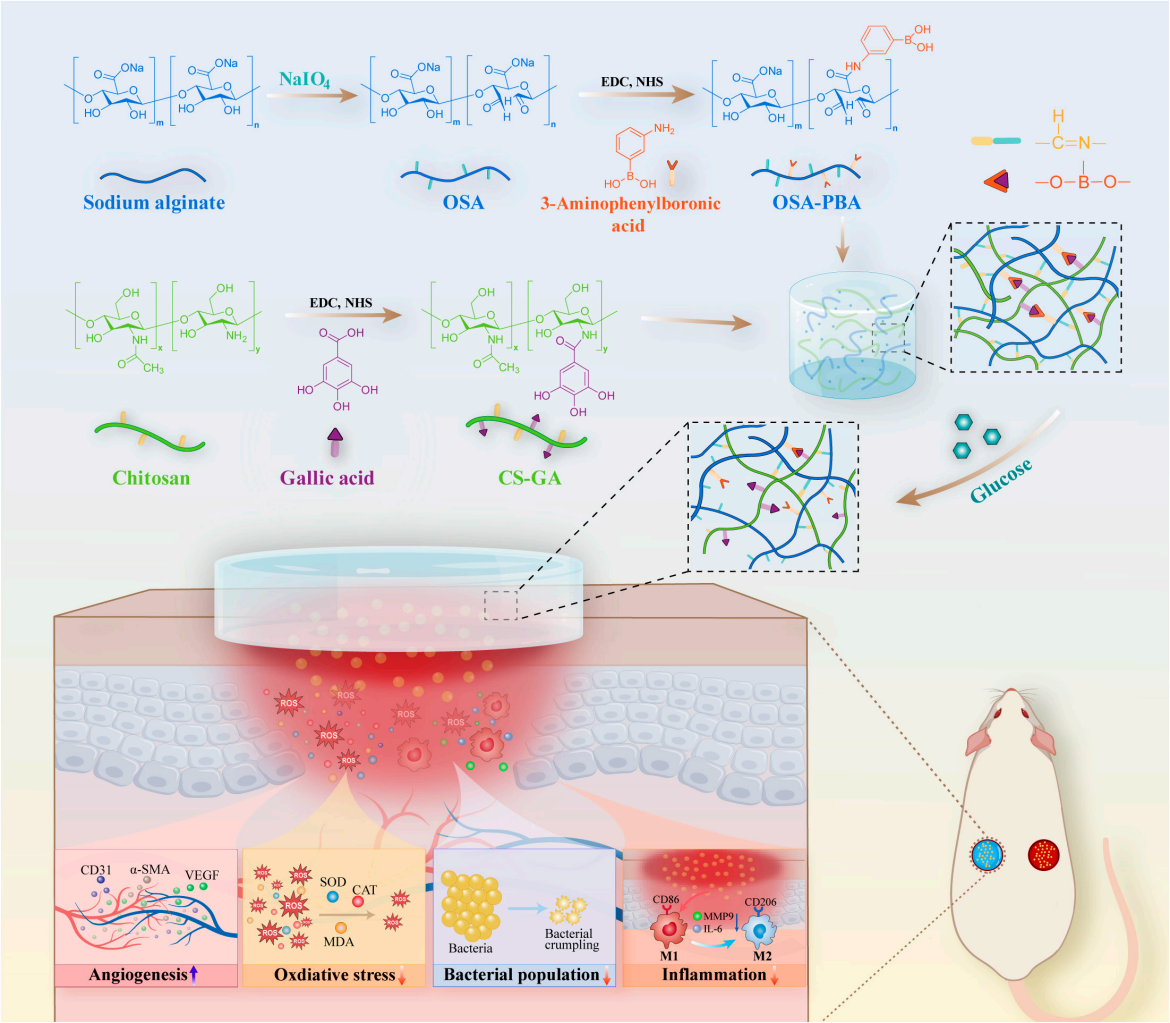
The synthesis process of phenylboronic-acid-functionalized oxidized sodium alginate and gallic-acid-conjugated chitosan (OSA-PBA/CS-GA) hydrogel and its multifunctional mechanisms in healing diabetic wounds. ROS, reactive oxygen species; SOD, superoxide dismutase; CAT, catalase; MDA, malondialdehyde; α-SMA, alpha smooth muscle actin; VEGF, vascular endothelial growth factor; IL-6, interleukin 6; MMP9, matrix metalloproteinase 9.

**Fig. 2.**
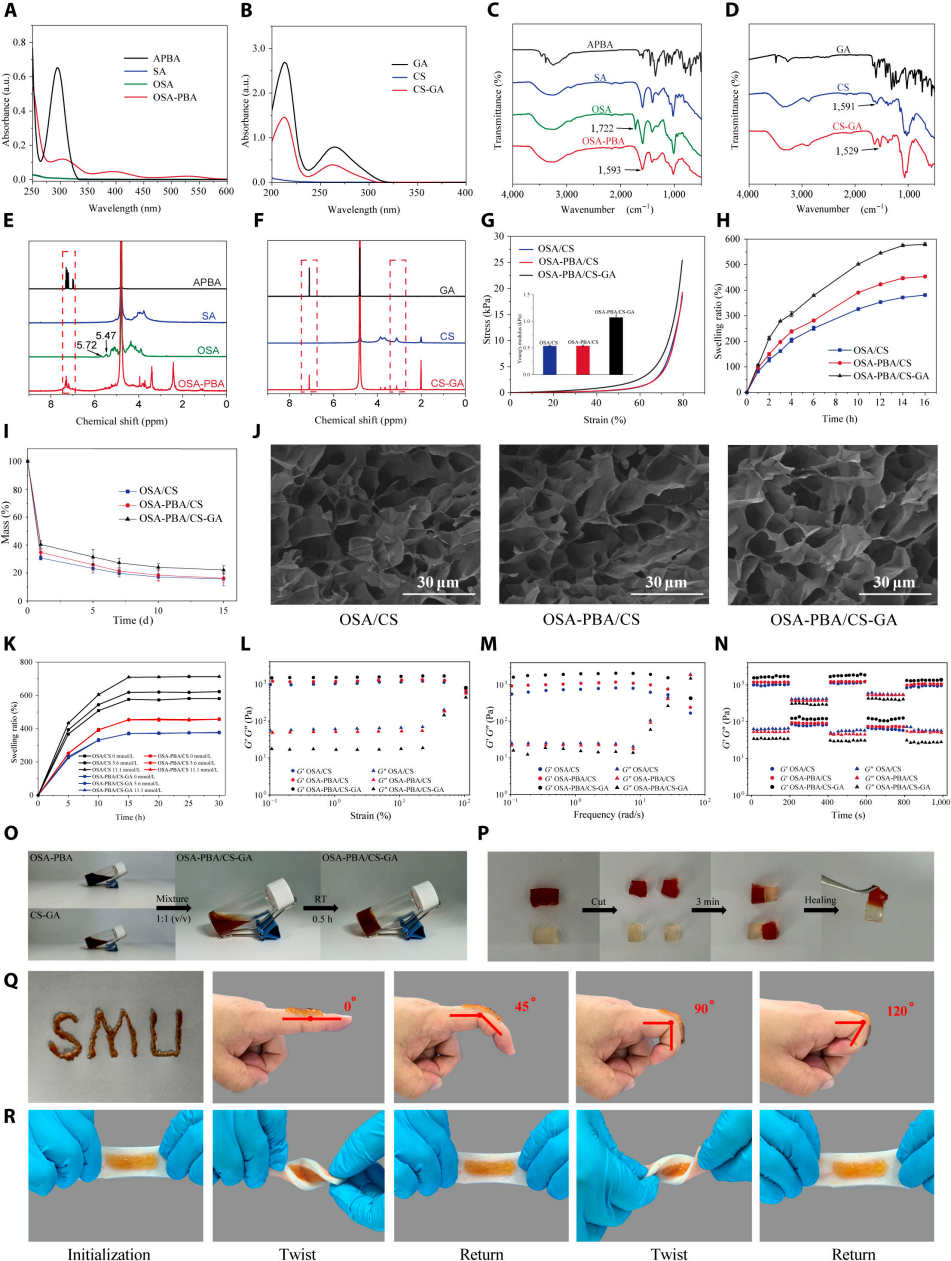
Preparation and characterization of OSA-PBA/CS-GA hydrogel. (A) Ultraviolet (UV) spectra of 3-aminophenylboronic acid (APBA), SA, OSA, and OSA-PBA; (B) UV spectra of GA, CS, and CS-GA; (C) Fourier-transform infrared (FTIR) spectra of APBA, SA, OSA, and OSA-PBA; (D) FTIR spectra of GA, CS, and CS-GA; (E) ^1^H nuclear magnetic resonance (NMR) spectra of APBA, SA, OSA, and OSA-PBA; (F) ^1^H NMR spectra of GA, CS, and CS-GA; (G) stress–strain curves of different hydrogels; (H) swelling rate curves of different hydrogels; (I) in vitro degradation profiles of different hydrogels; (J) microstructure of the hydrogels using scanning electron microscopy (SEM) analysis (scale bar: 30 μm); (K) swelling rate curves of hydrogels incubated in phosphate-buffered saline with glucose concentrations of 0, 5.6, and 11.1 mmol/L; (L–N) strain, frequency, and time sweep rheological curves; (O) preparation of OSA-PBA/CS-GA hydrogels; (P) hydrogel self-healing behavior; (Q) hydrogel injectability and its adaptation to skin twisting and physiological environments; (R) adhesion of the hydrogel. Bar graphs indicate mean ± SD (*n* = 3). RT, room temperature.

To construct a self-healing polysaccharide-based hydrogel with glucose-responsive release capability, PBA and GA were grafted onto OSA and CS molecular chains, respectively, through EDC/NHS-mediated amide coupling reactions (Fig. 1). UV spectroscopy was first performed to verify the successful incorporation of functional groups. APBA exhibited a characteristic absorption peak at 295 nm, whereas OSA-PBA showed a red-shifted peak at 303 nm, suggesting the introduction of APBA moieties into the OSA structure. CS-GA exhibited a characteristic absorption peak at 265 nm, corresponding to the π-electron transition of the GA benzene ring (Fig. 2A and B). The successful grafting of functional molecules was further confirmed by ^1^H nuclear magnetic resonance spectroscopy. Compared with OSA, OSA-PBA exhibited additional aromatic proton signals at *δ* = 7.3 to 7.6 ppm, corresponding to the phenyl groups of PBA, indicating successful incorporation of PBA moieties. For CS-GA, the characteristic peak at *δ* = 3.12 ppm was assigned to glucosamine residues of CS, while the newly appeared signal at *δ* = 7.10 ppm was attributed to aromatic protons of GA, confirming GA conjugation onto CS chains (Fig. 2E and F). Fourier-transform infrared analysis provided further evidence of successful modification. Compared with SA, OSA exhibited a new aldehyde C=O stretching peak at 1,722 cm^−1^, confirming oxidation of alginate. After PBA modification, characteristic peaks at 1,593 cm^−1^ (amide C=O stretching), 1,340 cm^−1^ (B–O stretching), and 707 cm^−1^ (phenyl C–H bending) appeared in OSA-PBA, indicating successful introduction of PBA groups through amide coupling. In CS-GA, the appearance of the benzene-ring C=C stretching peak at 1,529 cm^−1^ together with the decreased intensity of the –NH_2_ bending vibration at 1,591 cm^−1^ further confirmed GA conjugation (Fig. 2C and D).

SEM images revealed porous microstructures in all hydrogel formulations with different OSA-PBA/CS-GA ratios (Fig. S1A and Fig. 2J). Quantitative analysis based on ImageJ showed that the porosities of the 2:1 (OSA-PBA/CS-GA_1_), 1:1 (OSA-PBA/CS-GA_2_), and 1:2 (OSA-PBA/CS-GA_3_) groups were 50.59 ± 0.90%, 39.15 ± 1.41%, and 49.24 ± 0.52%, respectively (Fig. S1F). The effective crosslinking density (*ν*) was estimated according to the classical rubber elasticity theory using the plateau storage modulus (*G*′) obtained from frequency sweep measurements within the linear viscoelastic region, as expressed by *ν* = *G*′/*RT*, where *G*′ is the storage modulus, *R* is the universal gas constant (8.314 J mol^−1^ K^−1^), and *T* is the absolute temperature (310 K). The calculated crosslinking densities of the 2:1, 1:1, and 1:2 groups were 0.55 ± 0.02, 0.62 ± 0.01, and 0.57 ± 0.01 mol m^−3^, respectively (Fig. S1E). Rheological measurements showed that *G*′ remained consistently higher than *G*″ throughout the linear viscoelastic region, indicating the formation of a stable elastic hydrogel network with favorable mechanical stability. These dynamic mechanical properties are associated with the cooperative effect of the imine and boronate ester crosslinking network. Compression testing further revealed that the 1:1 formulation possessed relatively superior compressive modulus compared with the 2:1 and 1:2 groups. In addition, the equilibrium swelling ratios of the 2:1, 1:1, and 1:2 formulations in PBS (pH 7.4) were 628.30%, 579.62%, and 613.42%, respectively (Fig. S1G). Overall, the 1:1 formulation achieved the most balanced combination of mechanical performance, swelling behavior, and network stability. Therefore, it was selected as the optimal formulation for subsequent studies. Hereafter, the optimized 1:1 OSA-PBA/CS-GA_2_ hydrogel is abbreviated as OSA-PBA/CS-GA.

Swelling curves (Fig. 2H) showed that the swelling ratio of OSA-PBA/CS-GA gradually increased with time and differed from those of the OSA/CS and OSA-PBA/CS hydrogels. This swelling behavior is consistent with its porous microstructure and dual-dynamic covalent network. In vitro degradation was monitored over 15 d by measuring the mass loss of the hydrogels. As shown in Fig. 2I, the 3 formulations exhibited different degradation profiles. OSA-PBA/CS-GA displayed a moderate degradation rate, whereas OSA/CS and OSA-PBA/CS degraded more rapidly. Considering that DWs are typically exposed to a hyperglycemic microenvironment with fluctuating glucose levels, the swelling behavior of OSA-PBA/CS-GA was further evaluated under different glucose concentrations. As shown in Fig. 2K, all hydrogels reached swelling equilibrium within 15 h. The swelling ratio increased with glucose concentration, which is consistent with the reversible boronate ester interactions within the hydrogel network. Meanwhile, OSA-PBA/CS-GA maintained a balanced swelling behavior while exhibiting glucose-responsive swelling characteristics, which may be advantageous for DW applications.

Different release profiles were observed under glucose-free and glucose-containing conditions. The cumulative release tended to increase with increasing glucose concentration, with the release profile at 5.6 mmol/L glucose falling between those observed under glucose-free and 11.1 mmol/L glucose conditions (Fig. S1I). These results suggest that glucose modulates the release behavior of the hydrogel. Among the evaluated kinetic models (Table S1), the Weibull model provided the best fit for the release profiles in the presence of glucose, suggesting that glucose modulates the release kinetics of the hydrogel. In the present system, GA was covalently grafted onto CS through stable amide bonds via EDC/NHS-mediated coupling, and these amide linkages remain stable under physiological conditions as previously reported [29,30]. Therefore, the glucose-responsive behavior of the hydrogel is considered to arise from the reversible boronate ester interactions between PBA and galloyl groups. Under hyperglycemic conditions, glucose competitively interacts with PBA, shifting the dynamic boronate ester equilibrium and promoting the dissociation of reversible crosslinks, thereby loosening the hydrogel network and facilitating the release of galloyl-containing species. Since the release assay quantifies galloyl groups by UV absorbance, it cannot distinguish between CS-GA conjugates and free GA. Accordingly, the released species are inferred, based on the stability of the amide linkage and the hydrogel chemistry, to be predominantly CS-GA conjugates; however, their molecular identity has not been directly verified in the present study, and therefore this interpretation should be regarded as a mechanistic inference rather than direct experimental evidence.

The competitive interactions of glucose, GA, and OSA with PBA were evaluated using the ARS competitive binding assay (Fig. S1J). The experimentally determined equilibrium binding constants (*K*_eq_) showed that glucose exhibited a moderately higher binding affinity toward PBA than GA (63.9 and 32.3 M^−1^, respectively), whereas OSA displayed substantially weaker affinity. Although the higher affinity of glucose provides a thermodynamic basis for competitive binding, the ARS binding curves of glucose and GA largely overlapped within the physiological glucose concentration range (5 to 11.1 mmol/L) and became clearly distinguishable only at elevated glucose concentrations (20 to 50 mmol/L). These results suggest that glucose induces only limited competitive displacement of GA under physiological conditions and primarily modulates the dynamic boronate ester equilibrium rather than causing complete dissociation of the reversible boronate ester crosslinks. Accordingly, the proposed mechanism is based primarily on the experimentally determined equilibrium binding constants, whereas the aldehyde content of OSA-PBA is presented only as structural characterization of the material rather than evidence supporting competitive binding.

The stress–strain curves (Fig. 2G) displayed distinct mechanical behaviors among the hydrogel groups, with the optimized OSA-PBA/CS-GA hydrogel exhibiting superior compressive performance. Rheological characterization further verified its robust and stable elastic network. During the frequency sweep (Fig. 2M), both *G*′ and *G*″ exhibited minimal frequency dependence, suggesting a stable crosslinked architecture with reliable viscoelastic properties. The time sweep results (Fig. 2N) showed that *G*′ and *G*″ remained nearly constant throughout the measurement, demonstrating excellent long-term mechanical stability of the hydrogel network. Schiff base bonds form the fundamental framework and provide mechanical support owing to their relatively slow bond-exchange kinetics under physiological conditions, while boronate ester bonds endow the network with dynamic reversibility via fast bond-exchange behavior. Accordingly, the improved compressive performance of the OSA-PBA/CS-GA hydrogel stems from the combined contributions of both crosslinking types rather than a single-bond effect. Meanwhile, reversible boronate ester linkages dominate rapid-network rearrangement and endow the hydrogel with favorable injectability and self-healing capability [31–33].

In addition, the OSA-PBA/CS-GA hydrogel can be readily fabricated under ambient conditions via a simple and efficient gelation process (Fig. 2O). When a square hydrogel sample was cut into 4 fragments and brought into contact again (Fig. 2P), the separated pieces spontaneously reconnected and recovered a continuous structure, demonstrating excellent self-healing capability and the potential to recover a continuous structure after reconnection. In the injectability test (Fig. 2Q), the hydrogel was smoothly extruded through a 5-mL syringe equipped with a 26G needle, maintaining a continuous filamentous structure and allowing direct writing of the “SMU” pattern. Furthermore, the hydrogel adapted well to skin deformation at different twisting angles (0°, 45°, 90°, and 120°), indicating good injectability and adaptability to dynamic deformation under dynamic deformation (Videos S1 and S2). In the adhesion test (Fig. 2R), the hydrogel exhibited stable adhesion under complex physiological conditions and remained stably attached to porcine skin even under substantial torsional deformation. These favorable macroscopic properties are associated with the synergistic contribution of the dynamic covalent network formed by Schiff base and boronate ester bonds, which facilitates network rearrangement and structural recovery. Collectively, the OSA-PBA/CS-GA hydrogel exhibits excellent injectability and self-healing capability, providing a promising material platform for DW management.

### In vitro biocompatibility and pro-angiogenic performance of the OSA-PBA/CS-GA hydrogel

**Fig. 3.**
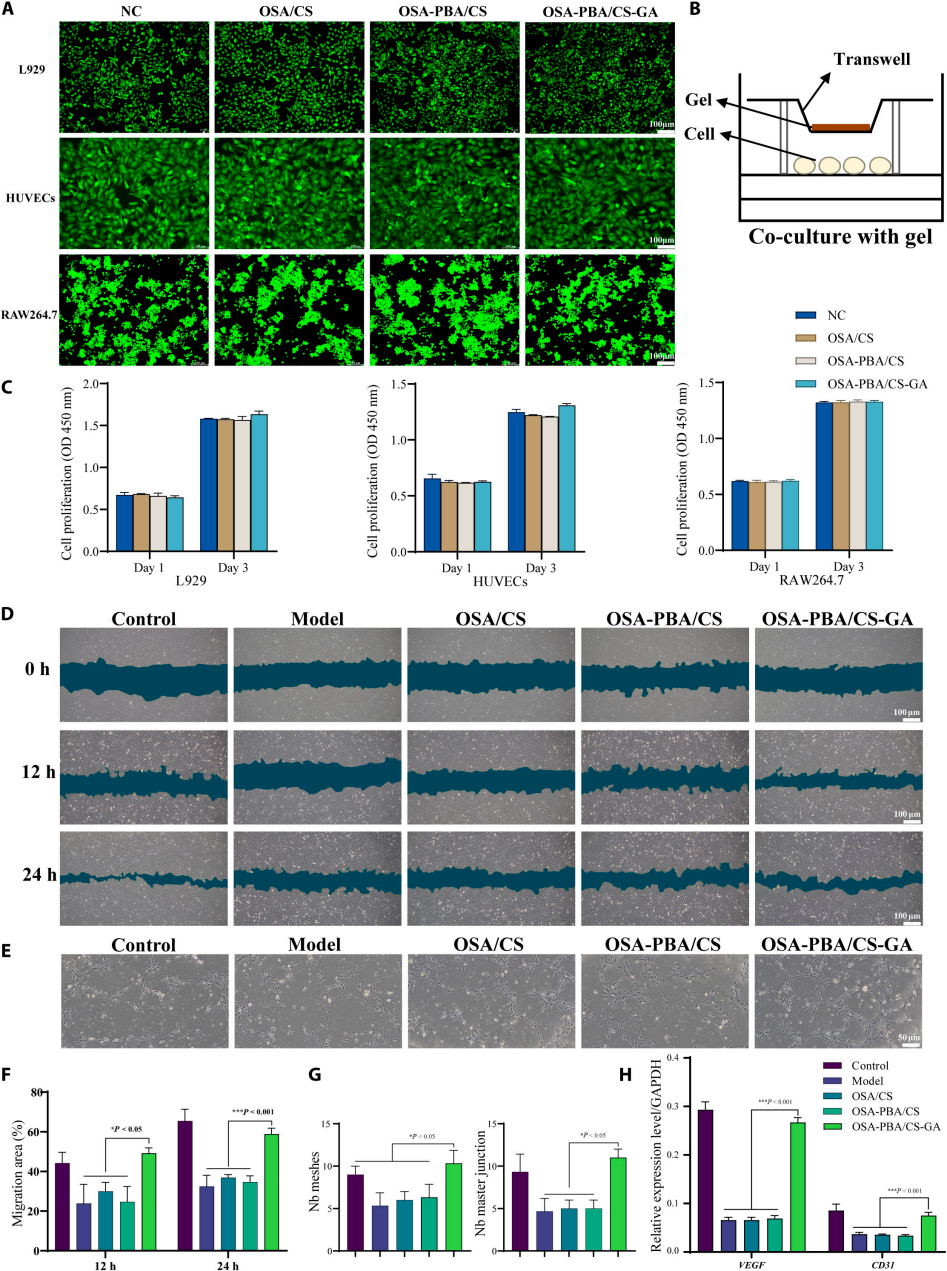
Biocompatibility and angiogenesis evaluation. (A) Live/dead staining for L929, human umbilical vein endothelial cells (HUVECs), and RAW264.7 cells at 72 h (scale bar: 100 μm); (B) sketch map for cell–gel co-culture using Transwell; (C) proliferation of L929, HUVECs, and RAW264.7 cells on day 1 and day 3, measured by Cell Counting Kit-8 assay and expressed as optical density (OD) values, with NC (negative control) representing untreated cells; (D) scratch assay images of HUVECs and (F) quantitative analysis of migration area rate at 12 h and 24 h (scale bar: 100 μm); (E) tube formation of HUVECs co-cultured with different hydrogels for 6 h (scale bar: 50 μm); (G) quantitative analysis of mesh number and master junction; (H) quantitative real-time polymerase chain reaction analysis of VEGF and CD31. GAPDH, glyceraldehyde-3-phosphate dehydrogenase. Bar graphs indicate mean ± SD (*n* = 3). ^*^P < 0.05, ^***^P < 0.001.

The biocompatibility and pro-angiogenic potential of the OSA-PBA/CS-GA hydrogel were evaluated through cell viability, migration, tube formation, and gene expression assays. Live/dead staining (Fig. 3A) showed abundant green (live) and almost no red (dead) fluorescence in L929, HUVECs, and RAW264.7 macrophages after 72 h of co-culture, indicating excellent cytocompatibility with negligible cytotoxicity. Quantitative Cell Counting Kit-8 assays (Fig. 3C) further indicated that all cell types maintained high proliferation rates for more than 3 d when exposed to hydrogel extracts, with no significant differences compared to the control group, supporting the material’s biosafety. Angiogenesis plays a key role in DWs’ healing. Chronic hyperglycemia in diabetes causes microvascular complications and impaired peripheral vascular function, resulting in inadequate perfusion, poor oxygenation, and limited nutrient delivery to the wound site [34]. In functional angiogenesis assessments, scratch assays (Fig. 3D and F) revealed that HUVECs co-cultured with OSA-PBA/CS-GA exhibited markedly enhanced migration, higher than the control and other hydrogel groups. Likewise, tube formation assays (Fig. 3E and G) showed that HUVECs formed more extensive capillary-like networks, with increased mesh number and master junctions in the OSA-PBA/CS-GA group, suggesting stronger stimulation of vascularization. VEGF and platelet endothelial cell adhesion molecule-1 (CD31) are key molecular markers involved in angiogenesis and microvessel density, playing central roles in DW repair [35]. Consistently, qRT-PCR analysis (Fig. 3H) showed up-regulated expression of CD31 and VEGF in HUVECs co-cultured with OSA-PBA/CS-GA hydrogel, supporting molecular-level activation of pro-angiogenic pathways. These results indicate that the OSA-PBA/CS-GA hydrogel not only maintains excellent biocompatibility but also actively promotes endothelial cell function, making it suitable for vascularized tissue regeneration in DW healing.

### In vitro antibacterial properties of OSA-PBA/CS-GA hydrogel

**Fig. 4.**
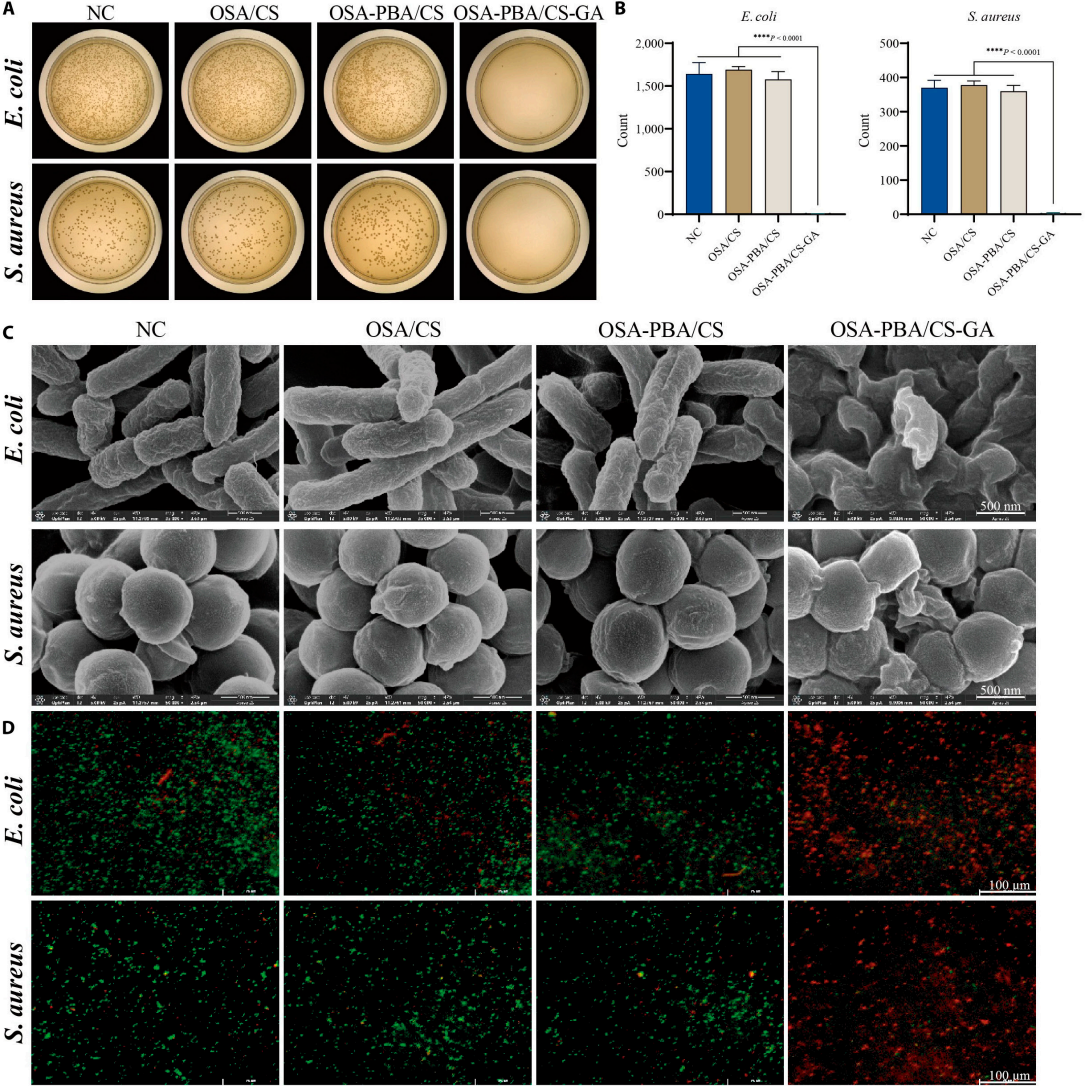
Antibacterial activity of OSA-PBA/CS-GA hydrogels against *Escherichia coli* and *Staphylococcus aureus* under 11.1 mmol/L glucose. (A) Photographs of bacterial colonies on Luria–Bertani agar plates; (B) quantitative analysis of bacterial counts; (C) SEM images showing bacterial morphology (scale bar: 500 nm); (D) live/dead fluorescence micrographs (green = live, red = dead) after treatment (scale bar: 100 μm). Bar graphs indicate mean ± SD (*n* = 3). ****P < 0.0001.

To evaluate the antibacterial activity of the OSA-PBA/CS-GA hydrogel, its effects against the representative DW pathogens *E. coli* and *S. aureus* were investigated. As shown in Fig. 4, colony images (Fig. 4A) and quantitative analysis (Fig. 4B) demonstrated that the OSA-PBA/CS-GA hydrogel markedly reduced bacterial colony formation compared with the control and the other hydrogel groups, indicating effective antibacterial activity against both gram-positive and gram-negative bacteria. SEM images (Fig. 4C) revealed severe morphological damage in bacteria treated with OSA-PBA/CS-GA, including membrane disruption, shrinkage, and cell deformation, suggesting that impairment of bacterial membrane integrity may contribute to its antibacterial activity. Live/dead staining (Fig. 4D) further supported these observations, with bacteria exposed to OSA-PBA/CS-GA exhibiting predominantly red fluorescence (dead cells), whereas bacteria in the control and single-component hydrogel groups were mainly stained green (live cells).

Comparative experiments under different glucose concentrations (Fig. S2) further demonstrated glucose-dependent antibacterial activity. At the physiological glucose concentration (5.6 mmol/L), the hydrogel already reduced bacterial colony formation, accompanied by partial membrane damage and an increased proportion of dead bacteria. Under hyperglycemic conditions (11.1 mmol/L), these antibacterial effects became more pronounced, as evidenced by further reductions in colony counts, more severe bacterial deformation, and increased bacterial death. These findings are consistent with the glucose-responsive release behavior of the hydrogel. The enhanced antibacterial activity is likely associated with the combined contributions of PBA, which can interact with bacterial cell surfaces [36], and the antimicrobial activity of galloyl groups [37,38]. Collectively, these results demonstrate that the OSA-PBA/CS-GA hydrogel effectively inhibits the growth of both Gram-positive and Gram-negative bacteria, highlighting its potential for preventing bacterial infection during DW healing.

### In vitro antioxidant and macrophage polarization regulation properties of OSA-PBA/CS-GA hydrogels

**Fig. 5.**
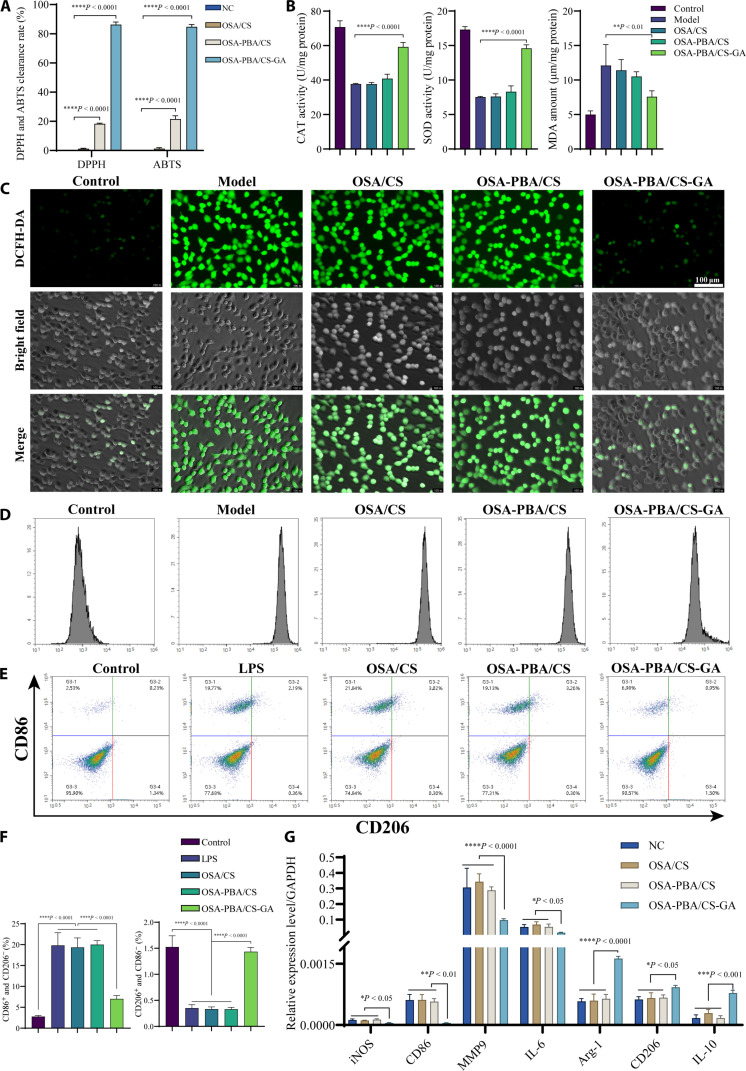
Antioxidant activity and macrophage polarization analysis of OSA-PBA/CS-GA hydrogels. (A) 2,2-Diphenyl-1-picrylhydrazyl (DPPH) and 2,2′-azino-*bis*(3-ethylbenzothiazoline-6-sulfonic acid (ABTS) free-radical-scavenging efficiency of OSA/CS, OSA-PBA/CS, and OSA-PBA/CS-GA hydrogels; (B) CAT activity, SOD-mimetic activity, and MDA content of the above 3 hydrogels; (C) ROS detection in cells via 2′,7′-dichlorodihydrofluorescein diacetate (DCFH-DA) analysis (scale bar: 100 μm); (D) flow cytometry histograms for ROS levels after DCFH-DA staining; (E) representative flow cytometry plots for CD86^+^/CD206^+^ macrophage polarization; (F) quantitative statistics of CD86^+^/CD206^+^ macrophage subsets; (G) relative mRNA levels of iNOS, CD86, MMP9, IL-6, Arg-1, CD206, and IL-10 in RAW264.7 cells with different treatments. Data are presented as mean ± SD (*n* = 3). *P < 0.05, **P < 0.01, ***P < 0.001, ****P < 0.0001. LPS, lipopolysaccharide.

At inflammatory sites, excessive ROS induce oxidative stress, leading to degradation of structural proteins, phospholipid membranes, enzymes, and DNA. This results in ECM destruction and cell death, severely compromising wound healing [39,40]. Therefore, antioxidant hydrogels may accelerate wound repair by effectively scavenging ROS and restoring redox balance [41]. As shown in Fig. 5A, the OSA-PBA/CS-GA hydrogel showed markedly enhanced free-radical-scavenging capacity compared to other groups, with 2,2-diphenyl-1-picrylhydrazyl and 2,2′-azino-*bis*(3-ethylbenzothiazoline-6-sulfonic acid radical clearance rates above 90% (^****^P < 0.0001), markedly higher than those of OSA/CS and OSA-PBA/CS. This is attributed to the *ortho*-trihydroxyphenyl structure of GA, which donates hydrogen atoms to neutralize ROS, scavenge free radicals, and halt oxidative chain reactions, thus protecting cells from oxidative damage [42]. Further biochemical assays (Fig. 5B) revealed that the hydrogel markedly increased CAT and SOD-mimetic activities while reducing MDA levels (^****^P < 0.0001, ^****^P < 0.0001, ^**^P < 0.01), demonstrating its ability to inhibit lipid peroxidation and protect cells against oxidative injury. At the cellular level, ROS scavenging was indicated by 2′,7′-dichlorodihydrofluorescein diacetate staining (Fig. 5C) and flow cytometry (Fig. 5D), which showed the weakest fluorescence intensity and lowest ROS levels in the OSA-PBA/CS-GA group, comparable to the normal control and clearly lower than the model group.

Macrophages are central to innate immunity and tissue repair during wound healing. Initially recruited as pro-inflammatory M1 cells, they eliminate pathogens and debris; subsequently, they shift toward a reparative M2 phenotype under healthy conditions [43]. However, in DWs, persistent M1 polarization and impaired M2 differentiation lead to chronic inflammation and delayed healing [44]. Accumulating evidence indicates that GA modulates inflammatory cytokine production and attenuates excessive immune activation, with GA-based hydrogels exerting anti-inflammatory effects primarily by suppressing nuclear factor kappa-B and Signal Transducer and Activator of Transcription 3 pathways [45]. To evaluate whether GA promotes M2 polarization, immunofluorescence staining was performed for CD86 and CD206. Flow cytometry (Fig. 5E and F) showed that the hydrogel effectively promoted macrophage polarization from M1 to M2, with a significant increase in M2 proportion (^****^P < 0.0001, ^****^P < 0.0001). Macrophages were co-stained for CD86 (green fluorescence) and CD206 (red fluorescence). Immunofluorescence staining results (Fig. S1K) revealed that OSA-PBA/CS-GA hydrogel markedly inhibited the expression of CD86 and effectively promoted the expression of CD206. These intuitive immunofluorescence findings were highly consistent with the quantitative data obtained from flow cytometry. Additionally, qRT-PCR analysis (Fig. 5G) provided further molecular evidence to support this conclusion; the expression levels of M1-related marker genes (*iNOS* and *CD86*) were markedly decreased, whereas the expression of M2-related marker genes (*Arg-1*, *CD206*, and *IL-10*) was notably up-regulated. Collectively, the above evidence indicates that the prepared hydrogel could efficiently promote macrophage polarization from pro-inflammatory M1 phenotype to anti-inflammatory M2 phenotype. In DWs, persistent overexpression of MMP9 disrupts ECM homeostasis, degrades growth factors, and sustains inflammation, contributing to impaired healing [46]. The OSA-PBA/CS-GA group exhibited markedly lower MMP9 expression relative to other treatment and control groups. Collectively, the OSA-PBA/CS-GA hydrogel shows strong antioxidant capacity and effectively regulates the immune microenvironment by promoting anti-inflammatory, pro-repair macrophage polarization, supporting the transition of DWs from chronic inflammation to tissue regeneration.

### In vivo amelioration of DW healing by OSA-PBA/CS-GA hydrogel

**Fig. 6.**
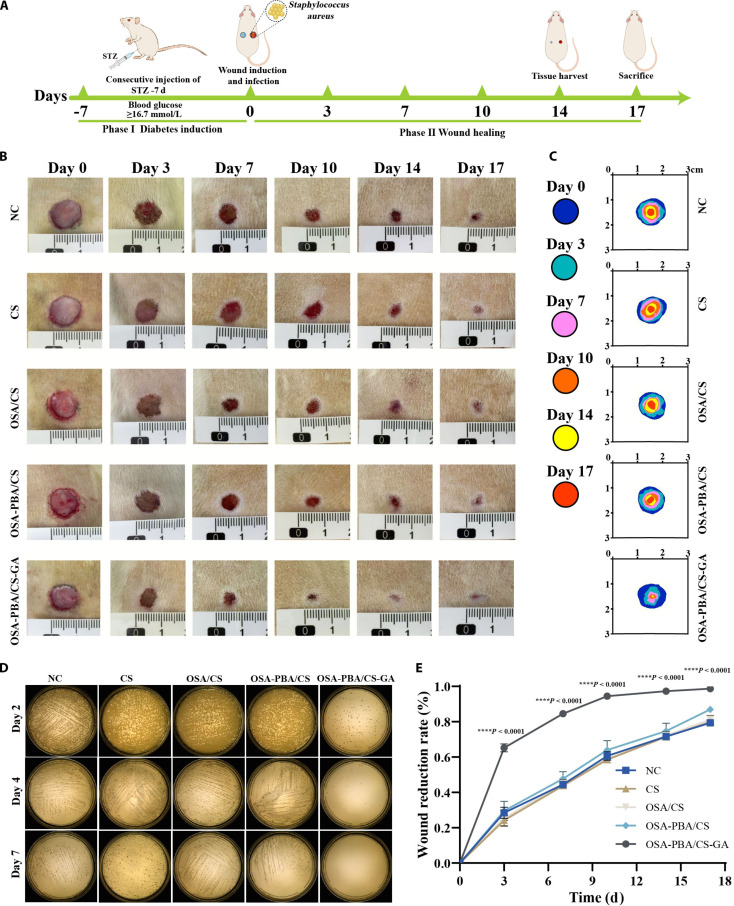
OSA-PBA/CS-GA hydrogels accelerate diabetic wound healing of streptozotocin (STZ)-exposed rats. (A) Schematic timeline for establishing the diabetic wound model, including STZ-induced diabetes (phase I) and wound infection/healing (phase II, days 0–17); (B) representative images showing infected wounds from different groups at 0, 3, 7, 10, 14, and 17 d following treatment; (C) superimposed contour images of wounds at days 0, 3, 7, 10, 14, and 17; (D) bacterial spread plate assay results of wound surface bacteria at days 2, 4, and 7; (E) statistical analysis of wound reduction rate over time. Data are presented as mean ± SD (*n* = 6). ****P < 0.0001.

To validate the in vivo effect of OSA-PBA/CS-GA hydrogel, an STZ-induced diabetic rat model combined with *S. aureus* infection was established (Fig. 6A), simulating pathological features of a DW. As shown in Fig. 6B, wounds treated with OSA-PBA/CS-GA exhibited markedly accelerated healing compared to control and single-component hydrogel groups. By day 17, nearly complete wound closure was observed in the OSA-PBA/CS-GA group, with minimal residual lesion area. Superimposed wound contour images (Fig. 6C) clearly illustrated progressive wound size reduction over time, demonstrating superior re-epithelialization and tissue repair. Bacterial burden on the wound surface was markedly reduced in the OSA-PBA/CS-GA group, with almost no detectable colonies by day 7 (Fig. 6D), indicating effective infection control in vivo, consistent with its strong antibacterial activity observed in vitro. Quantitative analysis of wound reduction rate (Fig. 6E) revealed that OSA-PBA/CS-GA achieved over 95% wound closure by day 17, significantly outperforming all other groups (^****^P < 0.0001). These results show that OSA-PBA/CS-GA not only eliminates pathogens but also creates a favorable microenvironment for healing through anti-inflammatory, antioxidant, and pro-regenerative actions. The excellent in vivo performance highlights its ability to overcome key barriers in DW repair, including persistent infection, impaired angiogenesis, and chronic inflammation, making it highly suitable for the clinical management of DWs. Additionally, visceral tissues were subjected to H&E staining to assess in vivo toxicity induced by OSA-PBA/CS-GA hydrogel. No significant pathological changes were noted in visceral tissue morphology across all groups, providing further evidence of excellent biocompatibility (Fig. S3).

### In vivo assessment of histological features and mRNA expression profiles of key molecular markers modulated by OSA-PBA/CS-GA hydrogel

**Fig. 7.**
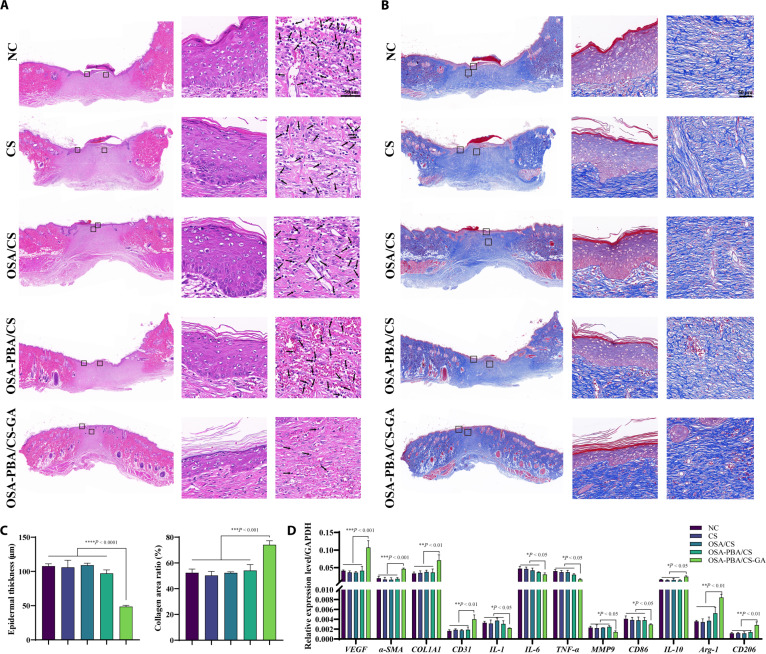
Evaluation of histological staining and relative mRNA expression levels of molecular markers. (A) Hematoxylin-and-eosin-stained tissues on day 14 (scale bar: 50 μm). Black arrows represent inflammatory cells; (B) Masson staining for tissues on day 14 (scale bar: 50 μm); (C) quantification of epidermal thickness and collagen area ratio; (D) relative expression of markers (α-SMA and COL1A1), angiogenic markers (VEGF and CD31), inflammatory cytokines (IL-1, IL-6, TNF-α, and MMP9), and macrophage markers (CD86, IL-10, Arg-1, and CD206). Data are presented as mean ± SD (*n* = 4). *P < 0.05, **P < 0.01, ***P < 0.001, ****P < 0.0001.

Histological analysis and gene expression profiling further revealed the superior tissue regeneration induced by the OSA-PBA/CS-GA hydrogel. Masson staining (Fig. 7B) showed that the OSA-PBA/CS-GA group exhibited denser and more organized collagen deposition than control and other treatment groups, indicating improved ECM synthesis and maturation. Consistently, H&E staining (Fig. 7A) revealed complete re-epithelialization, a thicker and well-organized epidermal layer, and reduced inflammatory cell infiltration in the OSA-PBA/CS-GA group by day 14, indicating accelerated tissue repair. In contrast, other treatment and control groups exhibited persistent inflammation with incomplete epithelial coverage, characteristic of chronic non-healing wounds. Quantitative analysis indicated a significant increase in both epidermal thickness and collagen area ratio (^****^P < 0.0001, ^***^P < 0.001), underscoring its ability to promote structural restoration of damaged skin (Fig. 7C). At the molecular level, qRT-PCR analysis (Fig. 7D) showed that OSA-PBA/CS-GA up-regulated key markers associated with tissue remodeling and angiogenesis, including α-SMA, COL1A1, VEGF, and CD31. Meanwhile, it efficiently suppressed pro-inflammatory cytokines (TNF-α, IL-1, and IL-6), indicating strong anti-inflammatory activity and protection against ECM degradation. Notably, the hydrogel promoted an anti-inflammatory macrophage phenotype, as evidenced by down-regulation of CD86 and up-regulation of IL-10, Arg-1, and CD206. From these findings, OSA-PBA/CS-GA not only accelerates histological repair but also orchestrates a favorable wound microenvironment by balancing inflammation, enhancing ECM deposition, and stimulating vascularization, key processes critical for high-quality healing in DWs.

### In vivo immune modulation for DW healing by OSA-PBA/CS-GA hydrogels

**Fig. 8.**
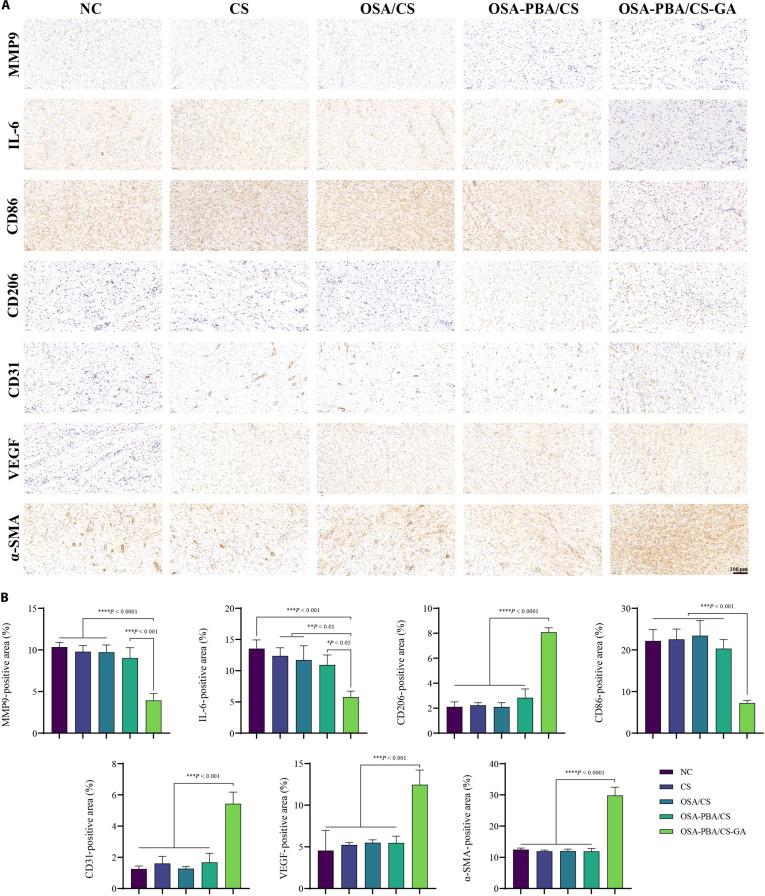
Expression analysis of MMP9, IL-6, CD86, CD206, CD31, VEGF, and α-SMA across groups. (A) Representative immunohistochemical images of diabetic wounds from each group, showing the distribution of MMP9, IL-6, CD86, CD206, CD31, VEGF, and α-SMA (scale bar: 100 μm); (B) quantification of positive staining area for each marker, reflecting expression levels. Data are presented as mean ± SD (*n* = 4). *P < 0.05, **P < 0.01, ***P < 0.001, ****P < 0.0001.

Immunohistochemistry was performed to evaluate in vivo expression and distribution of key inflammatory, immunomodulatory, and regenerative markers in wound tissues on day 14 (Fig. 8A). Quantitative analysis of positive staining areas (Fig. 8B) revealed that treatment with OSA-PBA/CS-GA markedly modulated the wound microenvironment compared to control groups. Specifically, the OSA-PBA/CS-GA group showed markedly reduced levels of pro-inflammatory mediators such as IL-6 and CD86, indicating effective suppression of chronic inflammation. In contrast, expression of the M2 macrophage marker CD206 was markedly up-regulated, indicating the hydrogel’s ability to promote polarization toward a reparative phenotype, consistent with findings from flow cytometry and qRT-PCR. Importantly, the OSA-PBA/CS-GA group showed strong positive signals for CD31 and VEGF, indicating robust angiogenesis and neovascularization. In DWs, impaired differentiation and function of α-SMA-positive myofibroblasts contribute to defective granulation tissue formation, reduced wound contraction, and delayed healing [47]. Immunohistochemical analysis revealed markedly enhanced α-SMA expression in the OSA-PBA/CS-GA group, indicating improved myofibroblast differentiation and maturation of granulation tissue, along with restored contractile capacity during repair. Sustained overexpression of MMP9 in DWs drives ECM degradation, growth factor inactivation, and persistent inflammation, impairing granulation tissue formation and re-epithelialization [48]. The OSA-PBA/CS-GA hydrogel alleviates the DW microenvironment by enhancing M2 polarization of macrophages, leading to the down-regulation of MMP9, thereby protecting the ECM and facilitating tissue regeneration. These results provide histological evidence that OSA-PBA/CS-GA coordinately regulates inflammation, promotes vascular regeneration, and accelerates tissue remodeling. The multimodal action further supports its application in comprehensive DW healing, bridging the gap between pathological dysregulation and functional restoration in chronic wounds.

## Conclusion

In summary, the OSA-PBA/CS-GA hydrogel represents a multifunctional biomaterial designed for DW repair. The dual-dynamic covalent network endows the hydrogel with favorable injectability, self-healing capacity, structural stability, and mechanical properties. Under hyperglycemic conditions, glucose-responsive release of galloyl-containing species contributes to the antioxidant, antibacterial, and anti-inflammatory functions of the hydrogel. Benefiting from these bioactivities, the hydrogel promotes M2 macrophage polarization, enhances angiogenesis, and accelerates wound closure in diabetic rats. Collectively, this study provides a facile strategy for constructing glucose-responsive polysaccharide-based hydrogels to ameliorate the pathological microenvironment of DWs, offering a promising platform for chronic wound management.

## Data Availability

Data will be made available on request.

## Supplementary Materials

Supplementary 1 Figs. S1 to S3 Table S1 Videos S1 and S2

## References

[1] Parveen K, Hussain MA, Anwar S, Elagib HM, Kausar MA. Comprehensive review on diabetic foot ulcers and neuropathy: Treatment, prevention and management. World J Diabetes. 2025;16(3): Article 100329. 40093290 10.4239/wjd.v16.i3.100329 PMC11885961

[2] Roy B. Pathophysiological mechanisms of diabetes-induced macrovascular and microvascular complications: The role of oxidative stress. Med Sci. 2025;13(3): Article 87. 10.3390/medsci13030087 PMC12286099 40700116

[3] Abd El-Khalik SR, Hafez YM, Elkholy RA. The role of circulating soluble fms-like tyrosine kinase-1 in patients with diabetic foot ulcer: A possible mechanism of pathogenesis via a novel link between oxidative stress, inflammation and angiogenesis. Microvasc Res. 2020;130: Article 103987. 32035919 10.1016/j.mvr.2020.103987

[4] Zhao S, Hu X, Zhao Y, Zhang Y, Jin Y, Hua F, Xu Y, Ding W. Hydrogel-based therapies for diabetic foot ulcers: Recent developments and clinical implications. Burns Trauma. 2025;13:tkae084. 39917278 10.1093/burnst/tkae084 PMC11801273

[5] Xiang T, Guo Q, Jia L, Yin T, Huang W, Zhang X, Zhou S. Multifunctional hydrogels for the healing of diabetic wounds. Adv Healthc Mater. 2024;13(1): Article e2301885. 37702116 10.1002/adhm.202301885

[6] Zheng BD, Xiao MT. Polysaccharide-based hydrogel with photothermal effect for accelerating wound healing. Carbohydr Polym. 2023;299: Article 120228. 36876827 10.1016/j.carbpol.2022.120228

[7] Zheng BD, Gan L, Tian LY, Chen GH. Protein/polysaccharide-based hydrogels loaded probiotic-mediated therapeutic systems: A review. Int J Biol Macromol. 2023;253(Pt 2): Article 126841. 37696368 10.1016/j.ijbiomac.2023.126841

[8] Sun X, Zhihui Q, Ye L, Zhang H, Yao F. Carbon nanotubes reinforced hydrogel as flexible strain sensor with high stretchability and mechanically toughness. Chem Eng J. 2020;382: Article 122832.

[9] Wang T, Yi W, Zhang Y, Wu H, Fan H, Zhao J, Wang S. Sodium alginate hydrogel containing platelet-rich plasma for wound healing. Colloids Surf B Biointerfaces. 2023;222: Article 113096. 36542954 10.1016/j.colsurfb.2022.113096

[10] Cai PF, Zheng BD, Xu YL, Li BX, Liu ZY, Huang YY, Ye J, Xiao MT. Multifunctional fish-skin collagen-based hydrogel sealant with dual-dynamic-bond cross-linked for rapid hemostasis and accelerated wound healing. Int J Biol Macromol. 2024;266(Pt 1): Article 131179. 38552698 10.1016/j.ijbiomac.2024.131179

[11] Wang H, Yang L, Yang Y. A review of sodium alginate-based hydrogels: Structure, mechanisms, applications, and perspectives. Int J Biol Macromol. 2025;292: Article 139151. 39725117 10.1016/j.ijbiomac.2024.139151

[12] Li X, Gong JP. Design principles for strong and tough hydrogels. Nat Rev Mater. 2024;9(6):380–398.

[13] Khalil AKA, Teow YH, Takriff MS, Ahmad AL, Atieh MA. Recent developments in stimuli-responsive polymer for emerging applications: A review. Results Eng. 2025;25: Article 103900.

[14] Ma R, Shi L. Phenylboronic acid-based glucose-responsive polymeric nanoparticles: Synthesis and applications in drug delivery. Polym Chem. 2014;5(5):1503–1518.

[15] Xiang Y, Xian S, Ollier RC, Yu S, Su B, Pramudya I, Webber MJ. Diboronate crosslinking: Introducing glucose specificity in glucose-responsive dynamic-covalent networks. J Control Release. 2022;348:601–611. 35714732 10.1016/j.jconrel.2022.06.016

[16] Xu Z, Liu G, Liu P, Hu Y, Chen Y, Fang Y, Sun G, Huang H, Wu J. Hyaluronic acid-based glucose-responsive antioxidant hydrogel platform for enhanced diabetic wound repair. Acta Biomater. 2022;147:147–157. 35649507 10.1016/j.actbio.2022.05.047

[17] Jin X, Lan W, Yong W, Yuan Z, Li X, Cui X. Application and development of stimulus-responsive hydrogel biomaterials for diabetic wound healing: A literature review. Front Med. 2025;12:1740559. 10.3389/fmed.2025.1740559 PMC12823876 41585269

[18] Al Zahrani NA, El-Shishtawy RM, Asiri AM. Recent developments of gallic acid derivatives and their hybrids in medicinal chemistry: A review. Eur J Med Chem. 2020;204: Article 112609. 32731188 10.1016/j.ejmech.2020.112609

[19] Deng Z, Guo Y, Wang X, Song J, Yang G, Shen L, Wang Y, Zhao X, Guo B, Wang W. Multiple crosslinked, self-healing, and shape-adaptable hydrogel laden with pain-relieving chitosan@borneol nanoparticles for infected burn wound healing. Theranostics. 2025;15(4):1439–1455. 39816695 10.7150/thno.102569 PMC11729554

[20] Han J, Meng Q, Xue S, Su W, Wu J. Silk fibroin methacryloyl hydrogel loaded with silver-gallic acid nanoparticles for enhanced diabetic wound healing. Int J Biol Macromol. 2025;307(Pt 4): Article 142108. 40089238 10.1016/j.ijbiomac.2025.142108

[21] Shapouri-Moghaddam A, Mohammadian S, Vazini H, Taghadosi M, Esmaeili SA, Mardani F, Seifi B, Mohammadi A, Afshari JT, Sahebkar A. Macrophage plasticity, polarization, and function in health and disease. J Cell Physiol. 2018;233(9):6425–6440. 10.1002/jcp.26429 PMC13482560 29319160

[22] Wolf SJ, Melvin WJ, Gallagher K. Macrophage-mediated inflammation in diabetic wound repair. Semin Cell Dev Biol. 2021;119:111–118. 34183242 10.1016/j.semcdb.2021.06.013 PMC8985699

[23] Tan WW, Lei SS, Long T, Xu ZL, Li DF, Mu CD, Ge LM. Polysaccharide-based injectable self-healing hydrogels with glucose-responsive drug release. Acta Polym Sin. 2023;54(8):1155–1165.

[24] Springsteen G, Wang B. Alizarin Red S as a general optical reporter for studying the binding of boronic acids with carbohydrates. Chem Commun. 2001;17(17):1608–1609. 10.1039/b104895n 12240405

[25] Brooks WLA, Deng CC, Sumerlin BS. Structure-reactivity relationships in boronic acid-diol complexation. ACS Omega. 2018;3(12):17863–17870. 31458380 10.1021/acsomega.8b02999 PMC6644144

[26] Feng M, Zeng X, Lin Q, Wang Y, Wei H, Yang S, Wang G, Chen X, Guo M, Yang X, et al. Characterization of chitosan-gallic acid graft copolymer for periodontal dressing hydrogel application. Adv Healthc Mater. 2024;13(7): Article e2302877. 38041691 10.1002/adhm.202302877

[27] Zhou Z, Deng T, Tao M, Lin L, Sun L, Song X, Gao D, Li J, Wang Z, Wang X, et al. Snail-inspired AFG/GelMA hydrogel accelerates diabetic wound healing via inflammatory cytokines suppression and macrophage polarization. Biomaterials. 2023;299: Article 122141. 37167893 10.1016/j.biomaterials.2023.122141

[28] Li H, Wei S, Ling Q, Wang R, Liu T, Yu H, Zhao P, Zhang K, Bian L, Liao W. Nanozyme-reinforced hydrogel spray as a reactive oxygen species-driven oxygenator to accelerate diabetic wound healing. Adv Mater. 2025;37(34): Article e2504829. 40492891 10.1002/adma.202504829

[29] Kahne D, Still WC. Hydrolysis of a peptide bond in neutral water. J Am Chem Soc. 1988;110(22):7529–7534.

[30] Radzicka A, Wolfenden R. Rates of uncatalyzed peptide bond hydrolysis in neutral solution and the transition state affinities of proteases. J Am Chem Soc. 1996;118(26):6105–6109.

[31] Liu Y, Liu Y, Wang Q, Han Y, Chen H, Tan Y. Doubly dynamic hydrogel formed by combining boronate ester and acylhydrazone bonds. Polymers. 2020;12(2): Article 487. 32098242 10.3390/polym12020487 PMC7077654

[32] Tang S, Ma H, Tu HC, Wang HR, Lin PC, Anseth KS. Adaptable fast relaxing boronate-based hydrogels for probing cell–matrix interactions. Adv Sci. 2018;5(9):1800638. 10.1002/advs.201800638 PMC6145256 30250802

[33] Terriac L, Helesbeux JJ, Maugars Y, Guicheux J, Tibbitt MW, Delplace V. Boronate ester hydrogels for biomedical applications: Challenges and opportunities. Chem Mater. 2024;36(14):6674–6695. 39070669 10.1021/acs.chemmater.4c00507 PMC11270748

[34] Brem H, Tomic-Canic M. Cellular and molecular basis of wound healing in diabetes. J Clin Invest. 2007;117(5):1219–1222. 17476353 10.1172/JCI32169 PMC1857239

[35] Li J, Chen J, Kirsner R. Pathophysiology of acute wound healing. Clin Dermatol. 2007;25(1):9–18. 17276196 10.1016/j.clindermatol.2006.09.007

[36] Zheng Y, Wang M, Zhang X, Wu Z, Gao L. A bacteria-responsive nanoplatform with biofilm dispersion and ROS scavenging for the healing of infected diabetic wounds. Acta Biomater. 2025;193:545–558. 39710222 10.1016/j.actbio.2024.12.042

[37] Tian Q, Wei S, Su H, Zheng S, Xu S, Liu M, Bo R, Li J. Bactericidal activity of gallic acid against multi-drug resistance *Escherichia coli*. Microb Pathog. 2022;173(Pt A): Article 105824. 36243382 10.1016/j.micpath.2022.105824

[38] Kiran K, Patil KN. Gallic acid inhibits *Staphylococcus aureus* RecA protein functions: Role in countering antibiotic resistance in bacteria. J Appl Microbiol. 2024;135(6): Article lxad227. 37804166 10.1093/jambio/lxad227

[39] Sindrilaru A, Peters T, Wieschalka S, Baican C, Baican A, Peter H, Hainzl A, Schatz S, Qi Y, Schlecht A, et al. An unrestrained proinflammatory M1 macrophage population induced by iron impairs wound healing in humans and mice. J Clin Invest. 2011;121(3):985–997. 21317534 10.1172/JCI44490 PMC3049372

[40] Wang G, Yang F, Zhou W, Xiao N, Luo M, Tang Z. The initiation of oxidative stress and therapeutic strategies in wound healing. Biomed Pharmacother. 2023;157: Article 114004. 36375308 10.1016/j.biopha.2022.114004

[41] Zhang X, Shu W, Yu Q, Qu W, Wang Y, Li R. Functional biomaterials for treatment of chronic wound. Front Bioeng Biotechnol. 2020;8:516. 32582657 10.3389/fbioe.2020.00516 PMC7283526

[42] Tang J. *Study on the antioxidant mechanisms of gallic acid and its ester derivatives: Molecular simulation and experiments*. Beijing (China): Beijing University of Chemical Technology.

[43] Kim SY, Nair MG. Macrophages in wound healing: Activation and plasticity. Immunol Cell Biol. 2019;97(3):258–267. 30746824 10.1111/imcb.12236 PMC6426672

[44] Koh TJ, DiPietro LA. Inflammation and wound healing: The role of the macrophage. Expert Rev Mol Med. 2011;13: Article e23. 21740602 10.1017/S1462399411001943 PMC3596046

[45] Huang H, Gong W, Wang X, He W, Hou Y, Hu J. Self-assembly of naturally small molecules into supramolecular fibrillar networks for wound healing. Adv Healthc Mater. 2022;11(12): Article e2102476. 35306757 10.1002/adhm.202102476

[46] Nissinen L, Kahari VM. Matrix metalloproteinases in inflammation. Biochim Biophys Acta. 2014;1840(8):2571–2580. 24631662 10.1016/j.bbagen.2014.03.007

[47] Hinz B, Phan SH, Thannickal VJ, Galli A, Bochaton-Piallat ML, Gabbiani G. The myofibroblast: One function, multiple origins. Am J Pathol. 2007;170(6):1807–1816. 17525249 10.2353/ajpath.2007.070112 PMC1899462

[48] Xue Y, Yang F, He Y, Wang F, Xia D, Liu Y. Multifunctional hydrogel with photothermal ROS scavenging and antibacterial activity accelerates diabetic wound healing. Adv Healthc Mater. 2025;14(6): Article e2402236. 39780538 10.1002/adhm.202402236

