# Supplementary material for: A Glucose-Responsive Hydrogel with Multifunctional Properties for Accelerated Diabetic Wound Healing
Source: Biomater Res. 2026 Aug 19;30:0405. doi: 10.34133/bmr.0405 (PMC13486732; doi:10.34133/bmr.0405)
Supplement: Supplementary file 1 [file bmr.0405.f1.zip › Supplementary materials.docx]

**Supplementary materials**

**Fig. S1** Characterization, glucose-responsive behavior, and immunoregulatory effect of OSA-PBA/CS-GA hydrogels. (a) SEM characterization of the three hydrogels (scale bar = 30 μm); (b) Rheological amplitude sweep test results; (c) Rheological frequency sweep test results; (d) Rheological time-dependent recovery test results; (e) Crosslinking density of different hydrogels; (f) Porosity of different hydrogels (g) Swelling ratio curves of different hydrogels; (h) Stress-strain curves of different hydrogels; (i) Glucose-responsive release profiles under different glucose concentrations; (j) Fluorescence displacement binding assay; (k) Immunofluorescence staining of macrophage polarization (CD86/CD206).

**Fig. S2** Antibacterial activity of OSA-PBA/CS-GA hydrogels. (a) Optical images showing bacterial colonies of *S. aureus* and *E. coli* after hydrogel treatment; (b) Quantitative analysis of bacterial counts; (c) SEM images showing bacterial morphology (scale bar: 500 nm); (d) Live/dead fluorescence micrographs (green = live, red = dead) after treatment (scale bar: 100 μm). Bar graphs indicate mean ± SD (n = 3). *****p* < 0.0001.

**Fig. S3** H&E-stained major organ tissue sections (lung, spleen, heart, liver, kidney) of experimental animals from different groups (NC, CS, OSA/CS, OSA-PBA/CS, OSA-PBA/CS-GA).

**Table S1** Sustained-release kinetics of galloyl-containing species from OSA-PBA/CS-GA hydrogels under different glucose conditions

| Kinetics model | Sustained-release equation in PBS containing  11.1 mmol/L glucose | Sustained-release equation in PBS containing  5.6 mmol/L glucose | Sustained-release equation in PBS |
| --- | --- | --- | --- |
| First-order | Q=100[1-exp(-0.1182t)]  (R^2^=0.8865 MSC=1.926) | Q=100[1-exp(-0.0304t)]  (R^2^=0.8760 MSC=0.600) | Q=100[1-exp(-0.0552t)]  (R^2^=0.5393 MSC=0.525) |
| Higuchi | Q=18.425t^0.5^  (R^2^=0.9533 MSC=2.813) | Q=14.090t^0.5^  (R^2^=0.8590 MSC=1.670) | Q=12.947t^0.5^  (R^2^=0.8927 MSC=1.982) |
| Korsmeyer-Peppas | Q=21.503t^0.4854^  (R^2^=0.9866 MSC=3.510) | Q=18.050t^0.4080^  (R^2^=0.9790 MSC=2.905) | Q=18.696t^0.3468^  (R^2^=0.9632 MSC=2.730) |
| Weibull distribution | Q=100[1-exp(-t0.6015/4.138)]  (R^2^=0.9957 MSC=4.888) | Q=100[1-exp(-t0.5000/25.400)]  (R^2^=0.9880 MSC=3.475) | Q=100[1-exp(-t0.4495/5.048)]  (R^2^=0.9785 MSC=3.270) |
